# Melatonin Mitigates Vitrification-Induced Cryoinjury in Mouse Embryos by Alleviating Metabolic Alterations

**DOI:** 10.3390/antiox15060667

**Published:** 2026-05-26

**Authors:** Pengyun Ji, Wenkui Ma, Mengmeng Zhao, Laiqing Yan, Yunjie Liu, Depeng Yin, Qianru Chen, Boda Chen, Hao Wu, Shuai Gao, Bingyuan Wang, Lu Zhang, Guoshi Liu

**Affiliations:** 1 State Key Laboratory of Animal Biotech Breeding, College of Animal Science and Technology, China Agricultural University, Beijing 100193, China; jipengyun@cau.edu.cn (P.J.); ma17610890927@163.com (W.M.); zhaomengmeng2023@cau.edu.cn (M.Z.); laiqingyan@cau.edu.cn (L.Y.); yunjieliu@cau.edu.cn (Y.L.); yindepeng01@163.com (D.Y.); chenqianru99@163.com (Q.C.); 18800160525@163.com (H.W.); gaoshuai1959@163.com (S.G.);; 2 Frontiers Science Center for Molecular Design Breeding, China Agricultural University, Beijing 100193, China

**Keywords:** melatonin, mouse morula, cryoinjury, embryo quality, mitochondrial function

## Abstract

Vitrification is a vital tool for the long-term preservation of animal genetic resources, yet cryoinjury—manifesting as oxidative stress, structural damage, and metabolic disorders—severely compromises its efficacy. Here, we investigated the protective effects of melatonin (MT) supplementation on the cryotolerance of mouse morulae. First, mouse morulae were assigned to four groups treated with vitrification and thawing media containing MT (0, 10^−3^, 10^−5^, and 10^−7^ M) to determine optimal MT concentration. Subsequently, embryos treated with the optimal MT concentration were evaluated for developmental competence, oxidative stress, apoptosis, and mitochondrial function. Furthermore, transcriptome sequencing was performed to elucidate MT-regulated molecular pathways. The results demonstrated that MT supplementation at 10^−5^ M significantly enhanced developmental competence, as evidenced by increased blastocyst rate, hatched blastocyst rate, total cell number and the inner cell mass (ICM)-to-total cell ratio compared to the MT-free group (*p* < 0.05). Consequently, embryo transfer outcomes showed higher live births and weaned pups in the 10^−5^ M MT group versus those in controls (*p* < 0.05), achieving levels comparable to fresh embryos (*p* > 0.05). Mechanistically, MT reversed cryoinjury-induced mitochondrial dysfunction by elevating membrane potential(MMP) and Adenosine Triphosphate(ATP) production while reducing Reactive Oxygen Species (ROS) accumulation (*p* < 0.05). Transcriptomic analysis further revealed that vitrification perturbed metabolic pathways, including amino acid/fatty acid degradation and glucose/pyruvate metabolism. MT downregulated cryoinjury-induced overexpression of *Rela* and *Nfkb1*, inhibiting excessive NF-κB activation and alleviating metabolic dysfunction. Additionally, MT restored expression of nucleotide synthesis genes (*Ctps2*, *Nme4*, *Gmps*, *Nudt2*, *Ppat*, *Impdh2*) critical for cell proliferation, and reversed downregulation of mitochondrial genes *Sucla2* and *Timm17a*, confirming restoration of mitochondrial homeostasis. In conclusion, melatonin alleviates vitrification-induced cryoinjury by restoring mitochondrial function, which rescues nucleotide synthesis and partially reverses associated metabolic dysfunction. These findings advance MT-mediated cryoprotection and underscore its translational value for embryo cryopreservation in animal genetic resource conservation.

## 1. Introduction

The cryopreservation of animal genetic material is essential for long-term genetic resource preservation, and an indispensable tool for distributing superior breeding traits, as well as a vital safeguard against the loss of biodiversity in endangered species [1,2]. Following the pioneering success of mouse embryo vitrification in 1985, this technology has undergone continuous refinement and optimization, expanding its application from rodents, such as mice and rats, to various large animal species [3,4]. Current embryo cryopreservation techniques are highly refined, enabling the successful vitrification of embryos at most embryo developmental stages. Due to its simplicity, speed, cost-effectiveness, and superior preservation efficacy, vitrification has become the preferred method in both clinical and research settings [5]. However, the vitrification process renders embryos highly susceptible to oxidative stress, leading to lipid peroxidation, membrane damage, and structural disruption—all of which impair post-thaw implantation and subsequent development [6]. Consequently, mitigating cryoinjury and enhancing cryopreservation efficiency remain critical priorities in the field.

In recent years, the search for cryoprotectants that offer optimal performance with minimal toxicity has emerged as a prominent area of investigation. Melatonin, an endogenous antioxidant, effectively alleviates oxidative stress and inhibits apoptosis, making it a promising candidate for use as a novel cryoprotectant. Recently, the impact of melatonin on embryonic development has been extensively characterized, with MT supplementation shown to enhance the developmental competence of both oocytes and embryos [7,8,9,10]. Melatonin supplementation significantly enhances in vitro embryonic development and increases embryo transfer success through its antioxidant, anti-apoptotic, and hormone-modulating effects, without compromising offspring health. Melatonin demonstrates beneficial effects on reproduction by improving embryo quality, implantation and pregnancy outcomes, likely through elevating serum estradiol and upregulating uterine melatonin receptors (MT1 and MT2) expression [11]. Melatonin also promotes in vitro embryonic development in mice by enhancing blastocyst rates and quality via antioxidant and anti-apoptotic mechanisms, such as upregulating SOD and Bcl-2 while downregulating p53 and Caspase-3 [8]. Furthermore, in sheep and bovines, melatonin treatment increased embryo recovery and improved pregnancy rates after embryo transfer, and in mice, embryos cultured with melatonin led to higher pregnancy rates and offspring survival upon transfer, all without adverse effects on offspring growth [7,8,12]. Its specific role in cryopreservation has also garnered significant attention, and numerous studies have demonstrated that incorporating melatonin into vitrification media improves the quality and subsequent developmental potential of vitrified–warmed oocytes in humans, cattle, and mice [13,14,15,16,17]. Melatonin’s emerging role as a novel protectant for embryo cryopreservation, and further study to standardize melatonin concentration and timing of supplementation to maximize efficacy while minimizing toxicity across different species and developmental stages are critical.

Mice serve as a well-established model to study the methods for vitrification of oocytes and embryos of other species due to the available of oocytes and embryos, as well as relatively straightforward in vitro evaluation parameters. To date, melatonin supplementation in cryopreservation has been restricted to pre-vitrification or post-thawed stages, with no reports yet documenting its direct supplementation in media for the whole process of embryo vitrification and thawing. Therefore, this study investigated the effects of melatonin on the vitrification and thawing of mouse embryos. By evaluating developmental competence, oxidative stress, apoptosis, and mitochondrial function of embryos experiencing vitrification, thawing and following in vitro culture, the protective effects and molecular mechanism underlying melatonin supplementation were determined. Our findings provide a practical strategy to enhance embryo preservation efficiency for the livestock industry and artificial reproduction clinics.

## 2. Materials and Methods

### 2.1. Animals and Embryo Collection

(1) Animals: Female ICR mice at eight weeks old and sexually mature male mice were purchased from Beijing Charles River Laboratory Animal Technology Co., Ltd., (Beijing, China). All mice were housed under conditions complying with the Standardization Administration of the People’s Republic of China (GB 14925-2023, Laboratory animal—Environment and housing facilities, Beijing, China). The experimental procedures involving mice were approved by the Animal Ethics Committee of China Agricultural University (Approval No. AW62306202-1-08).

(2) Collection of in vitro morulae: Pregnant mare serum gonadotropin (PMSG, Veterinary Drug Approval No. 110914564) and human chorionic gonadotrophin (hCG, Veterinary Drug Approval No. 110251282) were obtained from Ningbo Hormone Products Co., Ltd. (Ningbo, China). M2 medium (Cat. No. CE003) was purchased from M&C Technology, Beijing, China. KSOM medium (Cat. No. M1430) was purchased from Nanjing Aibei Biotechnology Co., Ltd., Nanjing, China. Female mice were intraperitoneally injected with 5 IU PMSG, and 48 h later, an injection of 5 IU hCG was performed. Subsequently, the female mice were caged with male mice. The next morning, female mice with vaginal plugs were selected and recorded as embryonic day 0.5 (E0.5). Twenty-four hours after hCG injection, mice were sacrificed, and their oviducts were excised and placed in pre-warmed M2 medium. The ampulla of the oviduct was punctured with a syringe to obtain zygotes. The isolated embryos were quickly transferred to M2 medium, rinsed at least three times, and then the morphologically normal embryos were selected and incubated in 60 µL KSOM culture drops. The culture drops were placed in a 60 mm Petri dish, which was prepared more than 2 h before incubation and equilibrated in an incubator (Model 3111, Thermo Fisher Scientific, Waltham, MA, USA) at 37 °C, 5% CO_2_, and saturated humidity. Following 60 h of in vitro culture, in vivo-derived pronuclear embryos developed into compacted morulae, which were then collected for the melatonin concentration screening.

(3) Collection of in vivo morulae: At 78–82 h post-hCG injection, the oviducts and uterine horns were isolated. The fimbriae of the oviduct were gently fitted onto a flushing needle under a microscope (Model SZ61, Olympus, Tokyo, Japan). The tip of the flushing needle was lightly pressed against the bottom of the Petri dish, and approximately 0.1 mL of M2 medium was used to flush the oviducts to collect morulae in vivo.

### 2.2. Experimental Design

(1) Screening of optimal melatonin concentration: MT (Cat. No. M5250) was purchased from Sigma-Aldrich (St. Louis, MO, USA). Mouse morulae produced in vitro were divided into five groups: Fresh group (untreated fresh in vitro morulae), and 0 M MT group (vitrification and thawing media only), and vitrification and thawing solution supplemented with melatonin at 10^−3^ M, 10^−5^ M, or 10^−7^ M. Except for the Fresh group, embryos in the other groups were treated with vitrification and thawing media containing melatonin but not vitrified in liquid nitrogen. Then, the embryos were cultured, and the outcomes of embryo development were recorded.

(2) Cryopreservation: Mouse morulae derived in vivo were divided into three groups, namely group F (fresh embryos), vitrification group (0 M MT), and V-MT group (vitrification thawing media supplemented with melatonin at optimal concentration). Embryos in the vitrification group and V-MT group were vitrified and then thawed. The developmental competence, oxidative stress, apoptosis, and mitochondrial function were compared between the treatment groups and fresh control morulae. Subsequently, single-embryo RNA sequencing (scRNA-seq) was conducted on blastocysts obtained after 24 h of in vitro culture of the morulae.

### 2.3. Embryo Vitrification and Thawing

(1) Preparation of Open Pulled Straw (OPS) and Vitrification Thawing Solutions: The OPSs were prepared according to the method described previously [18]. The middle part of a 0.25 mL plastic straw (Cat.No. 005565, I.V.M., L’ Aigle, France) was heated with an alcohol lamp and stretched to form OPS with a wall thickness of approximately 0.02 mm, an inner diameter of approximately 0.15 mm, an outer diameter of approximately 0.20 mm, and a thin end length of approximately 25 mm. Ficoll 70 (Cat. No. F2878), Sucrose (Cat. No. S-1888), Bovine Serum Albumin (BSA, Cat. No. A3311), Dimethyl sulfoxide (DMSO, Cat. No. D2650) and Ethylene Glycol (EG, Cat. No. 324558) were all purchased from Sigma-Aldrich (St. Louis, MO, USA). Dulbecco’s Phosphate-Buffered Saline (DPBS, Cat. No. 14190144), purchased from Thermo Fisher Scientific (China) Co., Ltd. (Shanghai, China). The pre-treatment solution contains 10% EG and 10% DMSO in DPBS medium. The vitrification medium EDFS35 consists of 17.5% (*v*/*v*) EG, 17.5% (*v*/*v*) DMSO, 30% (*w*/*v*) Ficoll and 0.5 mol/L sucrose dissolved in DPBS medium. The thawing solution is 0.5 mol/L sucrose solution.

(2) Vitrification of morulae: Vitrification of morulae was performed using the OPS vitrification procedure, with the specific modifications. Firstly, embryos were placed in the pre-treatment solution for 30 s, then transferred to the EDFS35 solution for 25 s. Subsequently, embryos were placed at the tip of the OPS and plunged into liquid nitrogen, with 5 to 8 embryos per straw. Then, the embryos were stored in liquid nitrogen for more than 10 min. All embryo manipulations were performed on a 37 °C constant-temperature stage.

(3) Morulae thawing: The straws were taken from liquid nitrogen, and then were immediately immersed in 0.5 mol/L sucrose solution pre-warmed to 37 °C. Embryos were expelled and incubated for 5 min, then washed three times in M2 medium before being transferred to pre-warmed KSOM medium for in vitro culture.

### 2.4. Detection of Reactive Oxygen Species (ROS) in Embryos

The hatched blastocysts, developed from the optimal melatonin concentration screening experiment, and thawed in vivo-derived morulae from the vitrification experiment were collected. The ROS levels in embryos were detected using the Reactive Oxygen Species Assay Kit (Cat. No. S0033S, Shanghai Beyotime Biotechnology Co., Ltd., Shanghai, China). For each group, 10 to 15 embryos were incubated in M2 medium containing 10 μmol/L 2′,7′-dichlorodihydrofluorescein diacetate (DCHFDA) in the dark for 30 min, then washed three times with DPBS-0.1% PVA. Polyvinyl Alcohol (PVA, Cat. No. P8136) was purchased from Sigma-Aldrich (St. Louis, MO, USA). Green fluorescence was excited and imaged under a fluorescence microscope (TE300, Nikon, Tokyo, Japan) with an excitation wavelength of 460 nm for ROS detection. Fluorescence images were saved in TIF format, and the fluorescence intensity of embryos was analyzed using Image J software (version 1.54).

### 2.5. Detection of Cell Proliferation

At 12 h post-thawing, the cell proliferation potential of blastocysts developed from vitrification–thawed morulae was examined via BeyoClick™ EdU-488 Cell Proliferation Assay Kit (Cat. No. S0027, Shanghai Beyotime Biotechnology Co., Ltd., Beijing, China). Briefly, EdU working solution (10 μmol/L) was prepared by diluting EdU 1:1000 in KSOM medium. Blastocysts developed 12 h post-thawing were incubated in EdU solution at 37 °C for 2 h, then washed twice with DPBS-0.1% PVA. Embryos were fixed in 4% paraformaldehyde (Cat. No. DF0135, Beijing Leagene Biotechnology Co., Ltd., Beijing, China) for 30 min, washed three times with DPBS-0.1% PVA, and permeabilized with 0.5% Triton X-100 (Cat. No. T9284, Sigma-Aldrich, St. Louis, MO, USA) for 30 min. After two washes, embryos were incubated in Click reaction solution at room temperature in the dark for 2 min, washed three times, stained with 4′,6-diamidino-2-phenylindole (DAPI, Cat. No. H-1200-1, Vector Laboratories Inc., Newark, CA, USA) for 5 min, and mounted on glass slides for fluorescence microscopy.The reagent components are shown in Table 1.

### 2.6. Embryo Transfer

On the same day when donor mice showed vaginal plugs, naturally estrous mice were caged with vasectomized male mice. On day 2.5, after the recipients were intraperitoneally injected with anesthetics (Avertin, 1.25%, Cat. No. MA0478, Meilunbio, Dalian, China) at a dose of 0.3 mL/100 g (*v*/*w*), a 1 mL syringe was used to puncture the uterine horn while avoiding blood vessels, and then a pre-pulled glass capillary was used to blow embryos into the uterine horn. Embryos of the Fresh group and melatonin-treated vitrification groups (0 M and 10^−5^ M MT) were transferred into recipient mice, with each recipient receiving 14 blastocysts matured for 12 h in vitro from cryopreserved–thawed in vivo morulae. On day 16 after transplantation, the number of offspring and their birth weight were observed and recorded. On day 21 after birth, their weaning weight was observed and recorded.

### 2.7. Detection of Cell Apoptosis (TUNEL Assay)

The apoptosis level of cells in blastocysts developed for 24 h in vitro from vitrificated-thawed morulae was determined using the One-Step TUNEL Apoptosis Assay Kit (Cat. No. S0033S, Shanghai Beyotime Biotechnology Co., Ltd., Shanghai, China). Briefly, embryos were washed three times in DPBS-0.1% PVA and fixed overnight at 4 °C in 4% paraformaldehyde solution. Embryos were then permeabilized in 0.5% Triton X-100-DPBS-0.1% PVA at room temperature for 1 h. Subsequently, embryos were transferred to TUNEL reaction medium and incubated at 38.5 °C in the dark for 1 h. After terminating the reaction, embryos were washed three times in DPBS-0.1% PVA, stained with DAPI for 5 min for nuclear staining, and then mounted on slides for total cell counting. Embryos were observed and imaged under a fluorescence microscope via TUNEL assay and DAPI staining, and the apoptosis rate of individual embryos was calculated. The apoptosis rate was calculated as follows: Apoptosis rate = (number of TUNEL-positive cells/total number of cells) × 100%.

### 2.8. Immunofluorescence Detection of Inner Cell Mass (ICM) and Trophoblast (TE) in Blastocysts

Blastocysts developed for 24 h in vitro from vitrificated-thawed morulae were collected, washed in DPBS-0.1% PVA, and treated with 0.5% pronase at 37 °C for 3–5 min to remove the zona pellucida. Embryos were fixed in 4% paraformaldehyde at room temperature for 1 h or 4 °C overnight, washed three times with DPBS-0.1% PVA, permeabilized with 0.5% Triton X-100 for 1-2 h, and blocked in 3% BSA for 1 h. For immunostaining, embryos were incubated overnight at 4 °C with Nanog antibody (Cat. No. 882, Santa Cruz Biotechnology, Inc., Santa Cruz, CA, USA; dilution: 1:200) and CDX2 antibody (Cat. No. MU392A-UC, BioGenex Laboratories, Inc., San Francisco, CA, USA; dilution ratio: 1:200), washed three times, then incubated with Alexa Fluor 488 and 647 secondary antibodies (Cat. Nos. Ab150113, Ab150077; Abcam, Cambridge, MA, USA; dilution: 1:1000 each) at room temperature for 1 h in the dark. After three washes, embryos were stained with DAPI for 5 min and mounted on glass slides. Blue fluorescence indicated total cells, green indicated trophectoderm (TE), and red indicated inner cell mass (ICM).

### 2.9. Determination of Mitochondrial Membrane Potential (MMP)

The mitochondrial membrane potential of vitrificated–thawed in vivo-derived morulae was detected using the JC-1 kit (Cat. No. C2006, Shanghai Beyotime Biotechnology Co., Ltd., Shanghai, China). The specific method was as follows: JC-1 was dissolved in M2 medium at a concentration of 10 μg/mL to prepare the working solution. Collected embryos were transferred to the prepared JC-1 M2 working solution and incubated at 37 °C for 15 min in the dark. Subsequently, embryos were washed three times with DPBS-0.1% PVA and observed and imaged under a fluorescence microscope. Image J software was used to analyze and quantify the green and red fluorescence intensities of each embryo, and the ratio of red fluorescence to green fluorescence was used as the mitochondrial membrane potential (MMP).

### 2.10. Detection of Adenosine Triphosphate (ATP) Content

The ATP content of morulae post-vitrification and thawing was determined using an Infinite F200 microplate reader. The ATP content of each sample well was calculated according to the measured ATP standard c measured using an ATP Assay Kit (Cat. No. S0027, Shanghai Beyotime Biotechnology Co., Ltd., Shanghai, China), following the manufacturer’s instructions. Embryos were treated with pronase to remove the zona pellucida, washed three times in M2 medium, and lysed in ATP lysis buffer. For each group, 5 morulae per replicate (3 replicates total) were lysed in 50 μL buffer by vortexing. ATP standards (0–32 μL, diluted to 1 μmol/L) and samples were added to a 96-well opaque microplate with 50 μL detection solution per well, incubated 5 min at room temperature, and measured using an Infinite F200 microplate reader (F200, Tecan, Männedorf, Switzerland). ATP content per embryo was calculated from the standard curve (Figure S1) by dividing total ATP by embryo number.

### 2.11. RNA-Seq and Enrichment Analysis

The thawed mouse morulae were further cultured in KSOM medium for 24 h. After collecting the blastocysts, the zona pellucida was removed with acidic Tyrode’s solution. Under a microscope, single embryos were transferred into 2 μL of cell lysis buffer using a mouth pipette. Amplification was performed using the Smart-Seq2 method. The data were analyzed according to our previous study [19]. Briefly, the raw sequencing reads were quality controlled using SOAPnuke software (v2.1.6), filtering out low-quality reads, adaptor contamination, and reads with more than 10% unknown bases (N). The quality-checked reads were aligned to the mouse reference genome using HISAT (default parameters) and mapped to the reference sequences using Bowtie2 to determine gene mapping rates. Gene and transcript expression levels were calculated using RSEM to obtain FPKM values. Differential expression analysis was performed with the DESeq2 package (v1.48.2) using default parameters, identifying significantly differentially expressed genes with q value < 0.001 and |log2 (Fold change)| ≥ 1. Gene enrichment analysis was conducted using ClusterProfiler, considering *p* < 0.05 in GO and KEGG pathways as significant. Time-series analysis of all DEGs was carried out using the fuzzy c-means algorithm in the Mfuzz package. A total of 1636 mouse transcription factors were downloaded from Animal TFDB 3.0 [20], and the intersection with differentially expressed genes identified differentially expressed transcription factors, which were visualized using a heatmap. Co-expression networks were constructed for genes with FPKM > 10 using the WGCNA package. The adjacency matrix was established by raising the co-expression measure (0.5 + 0.5 × correlation matrix) to the power of β = 16. Genes were clustered using a topological overlap matrix, and the correlation between two modules was determined based on module overlap.

### 2.12. Data Analysis

All data were statistically analyzed using SPSS 22.0 software, and graphs were generated using GraphPad Prism 9.0. The blastocyst rate, hatching blastocyst rate, and offspring birth rate were analyzed by the χ^2^ test (Chi-square test). Other data were analyzed by one-way analysis of variance (One-Way ANOVA). Normality and homogeneity of variance were tested before ANOVA. All data are expressed as the mean ± standard error (mean ± SE). Multiple comparisons among means were performed using Duncan’s multiple range test. A *p*-value < 0.05 was considered statistically significant, and *p* > 0.05 was considered non-significant. Statistical significance was denoted as follows: * (*p* < 0.05), ** (*p* < 0.01), and *** (*p* < 0.001).

## 3. Results

### 3.1. Determination of Optimal Melatonin Concentration for Mouse Embryo Vitrification

**Figure 1:**
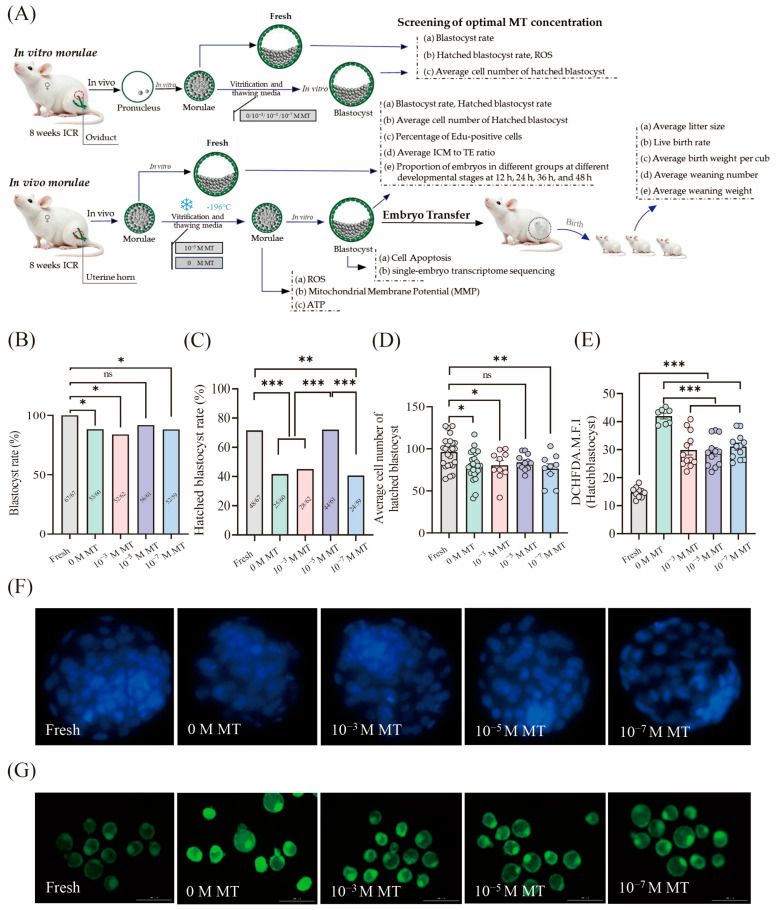
Effect of melatonin in vitrification and thawing media on mouse embryo in vitro development. (**A**) The experimental workflow. (**B**) Blastocyst rate (Fresh, *n* = 67; 0 M MT, *n* = 60; 10^−3^ M MT, *n* = 62; 10^−5^ M MT, *n* = 61; 10^−7^ M MT, *n* = 59). (**C**) Hatched blastocyst rate (Fresh, *n* = 67; 0 M MT, *n* = 60; 10^−3^ M MT, *n* = 62; 10^−5^ M MT, *n* = 61; 10^−7^ M MT, *n* = 59). (**D**) Average cell number of hatched blastocysts (Fresh, *n* = 25; 0 M MT, *n* = 24; 10^−3^ M MT, *n* = 10; 10^−5^ M MT, *n* = 12; 10^−7^ M MT, *n* = 9). (**E**) Quantification of ROS levels by analyzing the average 2′,7′-dichlorohydrofluorescein diacetate (DCHFDA) fluorescence intensity of each group of hatched blastocysts using Image J software (Fresh, *n* = 11; 0 M MT, *n* = 9; 10^−3^ M MT, *n* = 12; 10^−5^ M MT, *n* = 12; 10^−7^ M MT, *n* = 12). Scale bars: 200 µm. (**F**) Fluorescence map of the number of hatched blastocysts stained with DAPI. Scale bars: 50 µm. (**G**) ROS fluorescence images of hatched blastocysts from each group. * (*p* < 0.05), ** (*p* < 0.01), and *** (*p* < 0.001), ns: no significant difference.

Prior to the cryopreservation experiment, a preliminary screening was conducted to determine the optimal melatonin concentration (Figure 1A). Results revealed that the blastocyst rate of mouse morulae subjected to vitrification and thawing media without melatonin (0 M MT) was significantly lower than that of the Fresh group (Figure 1B, *p* < 0.05). Although supplementation of melatonin at various concentrations (10^−3^, 10^−5^, and 10^−7^ M) did not significantly alter blastocyst rates compared with the non-supplemented group (Figure 1B, *p* > 0.05), the hatching blastocyst rates in the 0 M, 10^−3^ M, and 10^−7^ M MT groups were significantly lower than those in both the Fresh group and the 10^−5^ M MT group (*p* < 0.05), whereas no significant difference was observed between the Fresh and 10^−5^ M MT groups (*p* > 0.05) (Figure 1C,F). The cell number in blastocysts in the 10^−5^ M MT group exhibited an upward trend and showed no difference with that in the Fresh group (*p* > 0.05) (Figure 1D).

Furthermore, analysis of ROS levels in hatched blastocysts revealed that cryoprotectant treatment significantly elevated embryonic ROS levels compared with the Fresh group (*p* < 0.05). Conversely, melatonin supplementation at all tested concentrations significantly attenuated ROS accumulation (*p* < 0.05); nevertheless, ROS levels in these groups remained significantly higher than those in the Fresh group (*p* < 0.05) (Figure 1E,G). These findings indicate that supplementation with 10^−5^ M melatonin improves the developmental competence of mouse embryos treated with vitrification and thawing media. These results suggest that melatonin functions as a partial scavenger of reactive oxygen species generated during vitrification and thawing procedures. Consequently, this concentration was selected for subsequent cryopreservation experiments.

### 3.2. Melatonin Restores Developmental Competence of Mouse Morulae Post-Vitrification and Thawing

**Figure 2:**
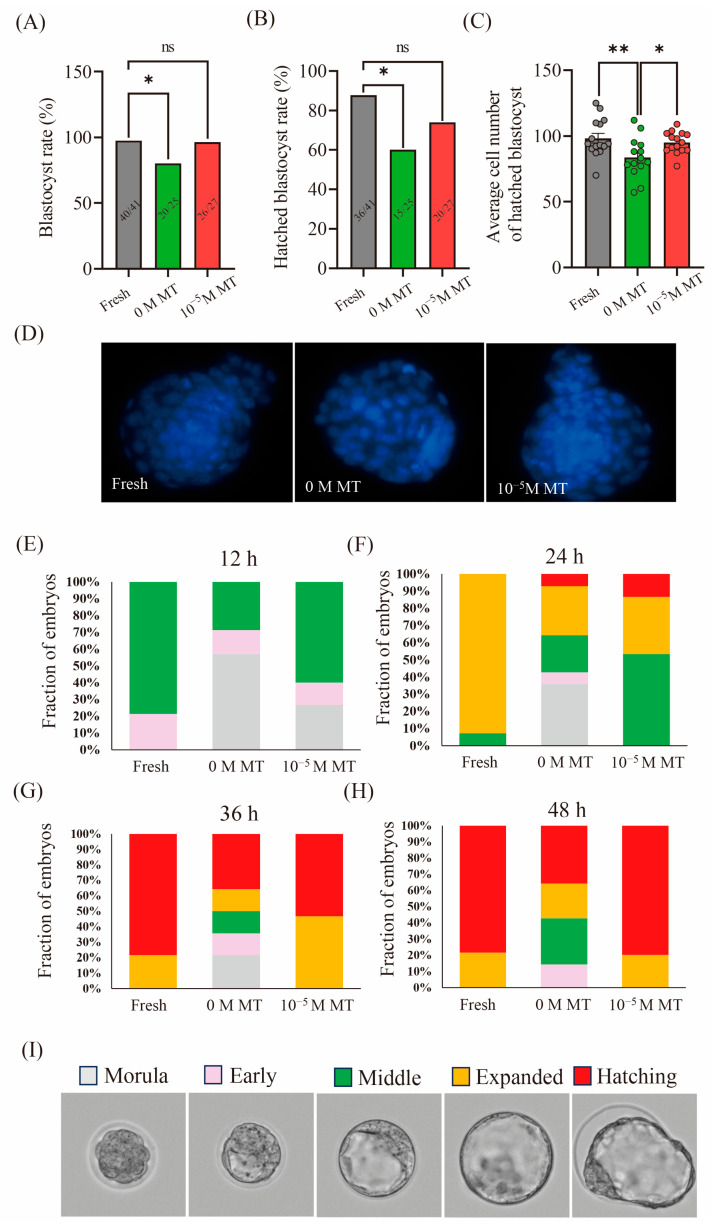
The effect of melatonin on the development of morulae in mice after vitrification and thawing. (**A**) Blastocyst rate (Fresh, *n* = 91; 0 M MT, *n* = 85; 10^−5^ M MT, *n* = 87). (**B**) Hatched blastocyst rate (Fresh, *n* = 91; 0 M MT, *n* = 85; 10^−5^ M MT, *n* = 87). (**C**) Average cell number of hatched blastocysts (Fresh, *n* = 15; 0 M MT, *n* = 14; 10^−5^ M MT, *n* = 15). (**D**) Fluorogram of hatched blastocysts stained with DAPI. Scale bars: 50 µm. (**E**–**H**) Proportion of embryos in different groups at different developmental stages at 12 h, 24 h, 36 h, and 48 h (Fresh, *n* = 15; 0 M MT, *n* = 14; 10^−5^ M MT, *n* = 15). (**I**) Pictures of representative embryos at each developmental stage. Scale bars: 50 µm. * (*p* < 0.05), ** (*p* < 0.01), ns: no significant difference.

Based on the screening results, 10^−5^ M melatonin was selected as the optimal concentration for subsequent vitrification of mouse morulae. The embryo recovery rate post-thawing was 100%. Compared with the Fresh group, the blastocyst rate in the 0 M MT group was significantly reduced (*p* < 0.05), whereas the 10^−5^ M MT group showed an increasing trend and was not significantly different from Fresh (*p* > 0.05) (Figure 2A). Similarly, the hatching blastocyst rate in the 0 M MT group was significantly lower than Fresh (*p* < 0.05), and there was no significant difference between the 10^−5^ M MT and Fresh groups (*p* > 0.05) (Figure 2B). The number of hatched blastocyst cells in the 0 M MT group was extremely significantly lower than that in the Fresh (*p* < 0.01) and 10^−5^ M MT groups (*p* < 0.05). Notably, no significant difference was observed between the 10^−5^ M MT and Fresh groups (Figure 2C,D). These results demonstrate that supplementing vitrification and thawing media with 10^−5^ M melatonin improves the developmental competence of vitrified and then thawed mouse morulae.

Embryo development was monitored for 48 h post-thaw using an automated intelligent imaging system (Figure 2I). As shown in Figure 2E–H, the developmental progression of the vitrified and then thawed mouse morulae lagged behind those in the Fresh group, particularly in the 0 M MT group, where morulae persisted at 36 h. However, at 36 and 48 h, the hatching blastocyst rate in the 10^−5^ M MT group was comparable to the Fresh group, and both were significantly higher than that in the 0 M MT group. The results indicate that 10^−5^ M melatonin supplementation accelerates post-thaw developmental progression in vitrified embryos.

### 3.3. Melatonin Enhances the Quality of Blastocysts Originated from Mouse Morulae Post-Vitrification and Thawing

**Figure 3:**
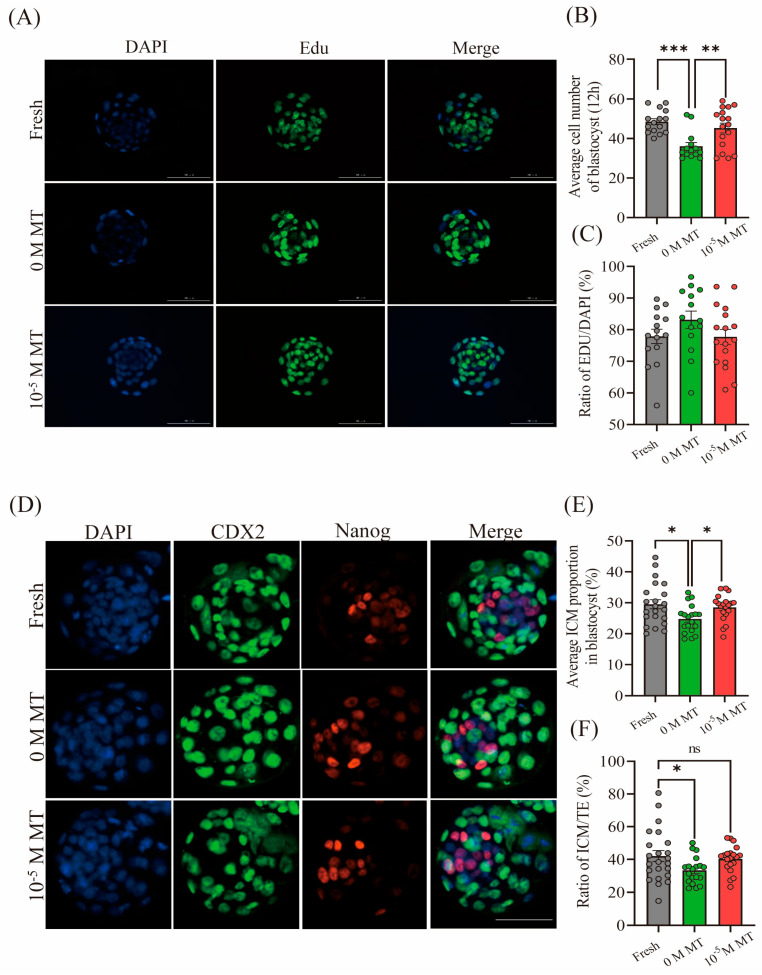
Effect of melatonin on the cell proliferation of blastocyst. (**A**) Cell proliferation of blastocysts in different treatment groups, as indicated by Edu staining on a scale of 100 μm. Green fluorescence indicates Edu-positive cells; blue fluorescence indicates DAPI (Fresh, *n* = 15; 0 M MT, *n* = 14; 10^−5^ M MT, *n* = 17). Scale bars: 100 µm. (**B**) Number of blastocysts developed from morulae for 12 h. (**C**) Percentage of Edu-positive cells. (**D**) The number of trophoblast cells (TEs) and inner cell mass cells (ICMs) in 24 h blastocysts of different treatment groups was indicated by staining with CDX2 and Nanog antibodies. Green fluorescence indicates CDX2 (TE), red fluorescence indicates Nanog (ICM), and blue fluorescence indicates DAPI (Fresh, *n* = 23; 0 M MT, *n* = 20; 10^−5^ M MT, *n* = 20). Scale bars: 50 µm. (**E**) Ratio of average ICM cell number to total cell number. (**F**) Average ICM to TE ratio. * (*p* < 0.05), ** (*p* < 0.01), and *** (*p* < 0.001), ns: no significant difference.

To evaluate whether MT supplementation improves the developmental potential of mouse morulae post-vitrification and thawing, the cell proliferation of blastocysts derived from morulae post-vitrification and thawing after 12 h in vitro culture was evaluated. It was found that the 0 M MT group had significantly fewer blastocyst cells than the Fresh and 10^−5^ M MT groups (*p* < 0.05), with no difference between the latter two (*p* > 0.05) (Figure 3A,B). EdU-positive cell proportions were similar across all groups (*p* > 0.05) (Figure 3C). These results indicate that 10^−5^ M MT enabled rapid recovery and proliferation of frozen embryos, while vitrification itself did not impair proliferative capacity. ICM and TE analysis by immunofluorescence staining showed that the 0 M MT group also had significantly lower ICM proportions (*p* < 0.05) (Figure 3D–F) and ICM:TE ratios compared to the Fresh group (*p* < 0.05); the 10^−5^ M MT group showed an increasing trend but no significant difference from the 0 M MT group (*p* > 0.05) (Figure 3F). Supplementing 10^−5^ M MT into vitrification and thawing media accelerated post-thaw development, improved cell proliferation and lineage allocation, and enhanced the developmental efficiency of the mouse morulae post-vitrification and thawing.

### 3.4. Effects of Melatonin on the Littering Size of Embryo Post Vitrification and Thawing

**Figure 4:**
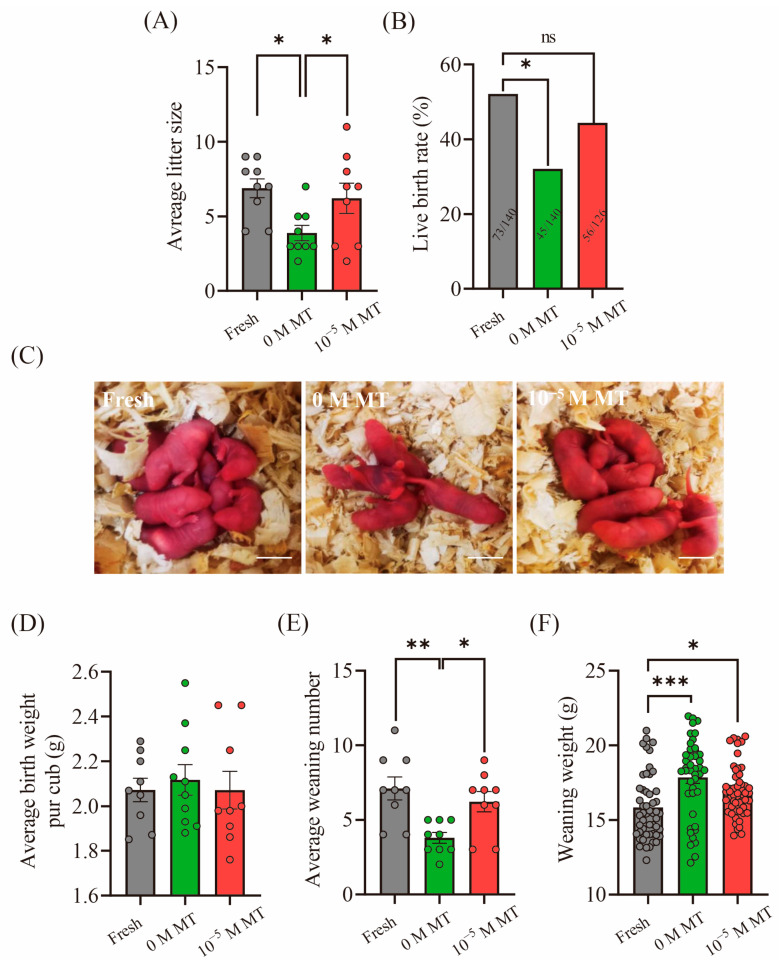
Effect of melatonin on litter size after transfer of mouse embryos post-vitrification and thawing. (**A**) Average litter size (Fresh, *n* = 10; 0 M MT, *n* = 10; 10^−5^ M MT, *n* = 9). (**B**) Live birth rate (Live birth rate = Total litter size/Total number of embryos transferred × 100%, 14 embryos transferred/recipient) (*n* = Litter size; Fresh, *n* = 73; 0 M MT, *n* = 45; 10^−5^ M MT, *n* = 56). (**C**) Image of a newborn mouse from embryo transfer. Scale bars: 1 cm. (**D**) Average birth weight per cub (*n* = Litter size; Fresh, *n* = 10; 0 M MT, *n* = 10; 10^−5^ M MT, *n* = 9; Average birth weight per cub = Litter weight/Litter size). (**E**) Average weaning number (Fresh, *n* = 10; 0 M MT, *n* = 10; 10^−5^ M MT, *n* = 9). (**F**) Average weaning weight (*n* = Number of weaning; Fresh, *n* = 71; 0 M MT, *n* = 39; 10^−5^ M MT, *n* = 56). * (*p* < 0.05), ** (*p* < 0.01), and *** (*p* < 0.001), ns: no significant difference.

To clarify whether melatonin supplementation in vitrification and thawing media improved the quality and developmental competence of thawed embryos in vivo, blastocysts developed 12 h after vitrification and thawing from the three groups were transferred into pseudo-pregnant female mice (Figure 4C). Average litter size (Figure 4A), live birth rate (Figure 4B), and average number of weaned pups (Figure 4E) in the 0 M MT group were significantly lower than those in the Fresh group (*p* < 0.05). The 10^−5^ M MT group showed significantly higher average litter size and weaned pups than those in the 0 M MT group (*p* < 0.05). The average birth rate in the 10^−5^ M MT group exhibited an increasing trend compared with the 0 M MT group, with no significant difference from the Fresh group (*p* > 0.05). No significant difference in average birth weight was observed among all groups (*p* > 0.05) (Figure 4D). The average weaning weight in the Fresh and MT group was significantly higher than that in the 0 M MT group (*p* < 0.05) (Figure 4F). Vitrification and thawing deteriorated embryo quality, as evidenced by reduced litter size and birth rate, while MT reversed these defects.

### 3.5. Melatonin Preserves Mitochondrial Integrity and Scavenges ROS in Mouse Morulae Post-Vitrification and Thawing

**Figure 5:**
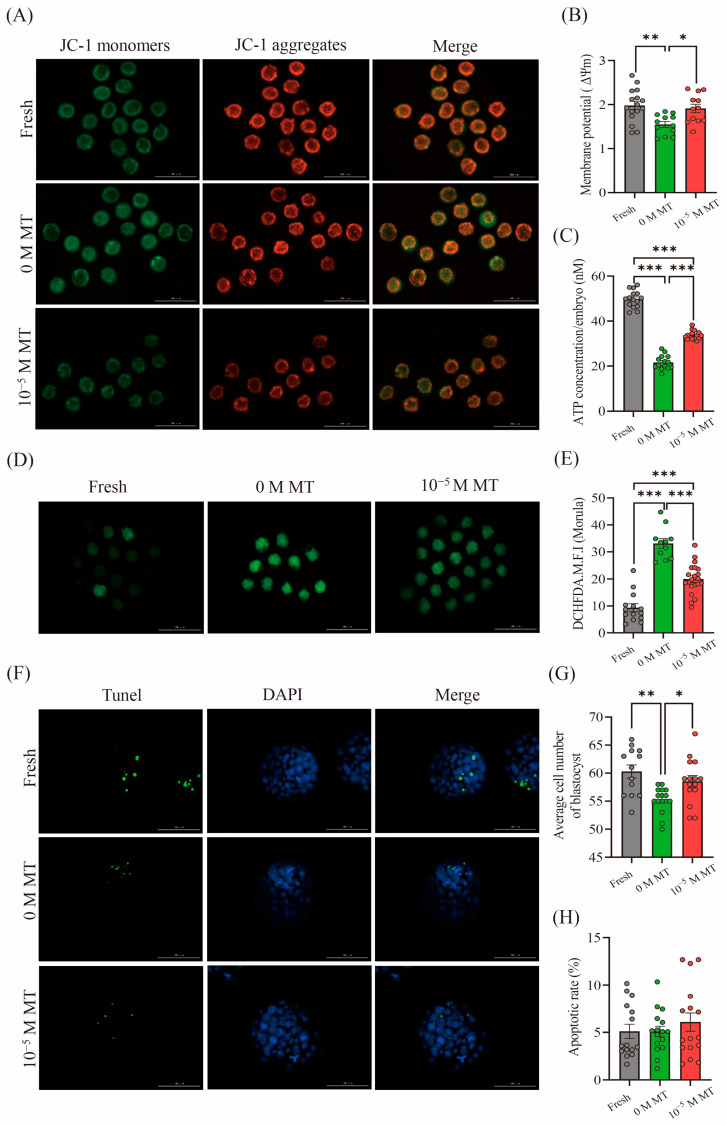
Effect of melatonin on mitochondrial function and cell apoptosis in mouse morulae post-vitrification and thawing. (**A**) JC-1 staining images of mitochondrial membrane potential in morulae, red fluorescence indicates high MMP and green indicates low MMP. Scale bars: 200 µm. (**B**) Statistics of MMP (Fresh, *n* = 16; 0 M MT, *n* = 12; 10^−5^ M MT, *n* = 12). (**C**) ATP concentration/embryo (Fresh, *n* = 15; 0 M MT, *n* = 15; 10^−5^ M MT, *n* = 15). (**D**) ROS level in morulae. Scale bars: 200 µm. (**E**) Quantification of ROS levels by Image J (Fresh, *n* = 15; 0 M MT, *n* = 11; 10^−5^ M MT, *n* = 22). (**F**) TUNEL staining. Green, TUNEL positivity indicates cells undergoing apoptosis; blue, DAPI fluorescent indication of nuclei. Scale bars: 100 µm. (**G**) Average cell number of blastocyst (Fresh, *n* = 13; 0 M MT, *n* = 16; 10^−5^ M MT, *n* = 15). (**H**) Apoptosis rate (Fresh, *n* = 15; 0 M MT, *n* = 16; 10^−5^ M MT, *n* = 16). * (*p* < 0.05), ** (*p* < 0.01), and *** (*p* < 0.001).

To investigate the mechanism by which melatonin (MT) supplementation exerts protective effects from vitrification, the embryonic mitochondrial function, oxidative damage and apoptosis level were evaluated. Post-vitrification and thawing, the mitochondrial membrane potential (MMP) of morulae in the 0 M MT group was significantly decreased compared with that in the Fresh group (*p* < 0.01) (Figure 5A,B), which was restored by melatonin treatment. The 10^−5^ M MT group showed significantly higher membrane potential than the 0 M MT group, with no significant difference from the Fresh group. Similarly, ATP content in embryos of the 0 M MT group was significantly lower than the Fresh group (*p* < 0.05), which was also elevated by 10^−5^ M MT (*p* < 0.05), but remained significantly lower than the Fresh group (*p* < 0.05) (Figure 5C). These results indicate that vitrification impairs mitochondrial function, and 10^−5^ M MT supplementation alleviates this damage.

Then, the ROS levels in morulae post-vitrification and thawing were detected by DCFHDA fluorescence intensity (Figure 5D,E), which were significantly higher in the 0 M MT group than the Fresh group. After 10^−5^ M MT supplementation, ROS levels were significantly reduced (*p* < 0.05), but remained significantly higher than the Fresh group (*p* < 0.05) (Figure 5E). This suggests that vitrification causes oxidative damage, and 10^−5^ M MT partially scavenges reactive oxygen species generated during vitrification. The apoptosis level of blastocysts, matured for 24 h in vitro from morulae post-vitrification and thawing, was determined. The number of blastocyst cells in the 0 M MT group was significantly lower than the Fresh and 10^−5^ M MT groups (*p* < 0.05), with no significant difference between the latter two (*p* > 0.05) (Figure 5F,G). TUNEL staining showed similar apoptosis rates across all three groups (*p* > 0.05) (Figure 5H), indicating that cryopreservation and MT supplementation do not affect embryonic apoptotic levels. Cryopreservation impairs mitochondrial function and causes oxidative damage in mouse embryos. Supplementing 10^−5^ M MT into vitrification and thawing media alleviates mitochondrial dysfunction and reduces oxidative stress, thereby improving blastocyst development.

### 3.6. Melatonin Rescues Vitrification-Induced Transcriptomic Changes in Mouse Embryos

**Figure 6:**
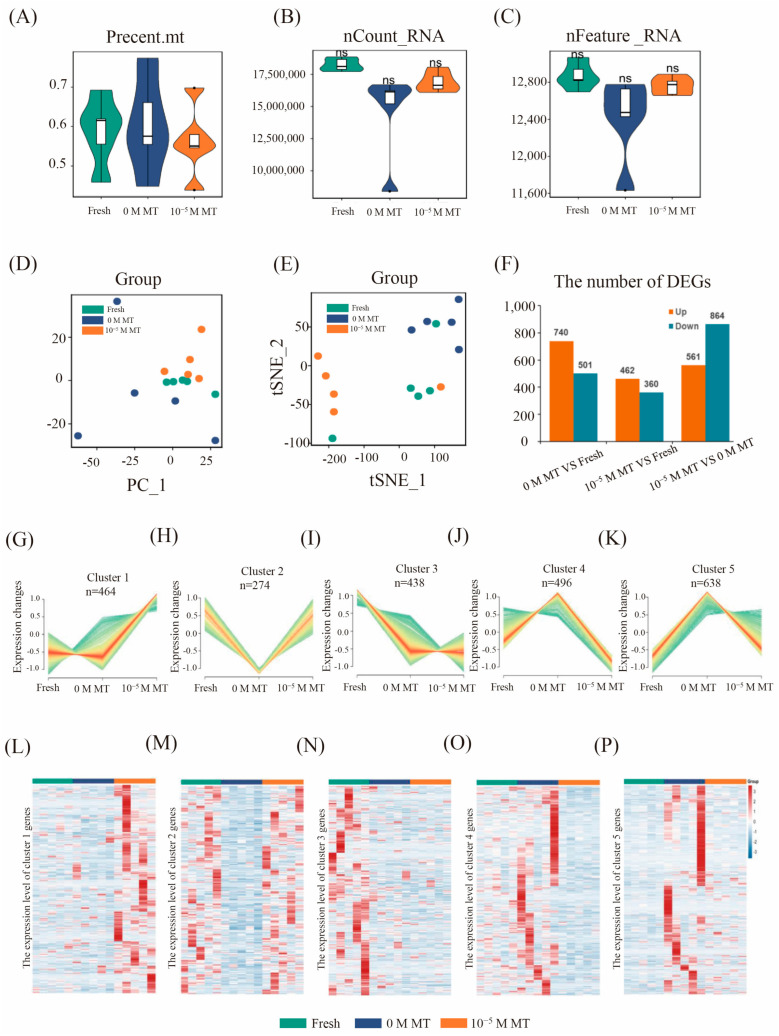
Effect of melatonin on gene expression in mouse embryos post-vitrification and thawing. (**A**) Violin plots showing the proportion of mitochondrial genes in samples across different groups. (**B**) Violin plots of nCount RNA in samples across different groups. (**C**) Violin plots of nFeature RNA in samples across different groups. (**D**) PCA plots of samples across different groups. (**E**) tSNE plots of samples across different groups. (**F**) Bar chart showing the distribution of differentially expressed genes. (**G**–**K**) Analysis of the expression patterns of DEGs, Each line shows the average expression level of one gene from all samples. (**L**–**P**) Heat maps showing the expression levels of each gene across different clusters. The color gradient from blue to red indicates the average gene expression levels from low to high, ns: no significant difference.

To investigate the effects of vitrification and melatonin supplementation on gene expression in mouse embryos, single-embryo transcriptome sequencing was performed. Firstly, mitochondrial content in all 15 samples was below 5% (Figure 6A), with no significant differences in total RNA count or functional RNA count among groups (*p* > 0.05, Figure 6B,C). PCA and t-SNE analysis showed no significant overall transcriptional differences among groups (Figure 6D,E). DESeq2 analysis identified 2310 significantly differentially expressed genes (DEGs) (*p* < 0.05) (Figure 6F) between Fresh and 0 M MT (740 upregulated, 501 downregulated), and 0 M MT vs. 10^−5^ M MT (561 upregulated and 864 downregulated). Mfuzz clustering revealed five distinct expression patterns: Cluster 1 (464 genes) highly expressed in 10^−5^ M MT group; Cluster 2 (274 genes) lowly expressed in 0 M MT group but similar to Fresh in 10^−5^ M MT group, indicating melatonin-mediated cryoinjury alleviation; Cluster 3 (438 genes) lowly expressed in both frozen groups, suggesting cryoinjury-related expression; Clusters 4 (496 genes) and 5 (638 genes) both highly expressed in 0 M MT group (*p* < 0.05) (Figure 6G–P). These results indicate that vitrification significantly alters gene expression, while melatonin supplementation partially rescues these transcriptional changes through specific gene clusters associated with cryoinjury.

### 3.7. Melatonin Rescues Metabolic and Immune Pathways in Vitrified and Thawed Mouse Embryos

**Figure 7:**
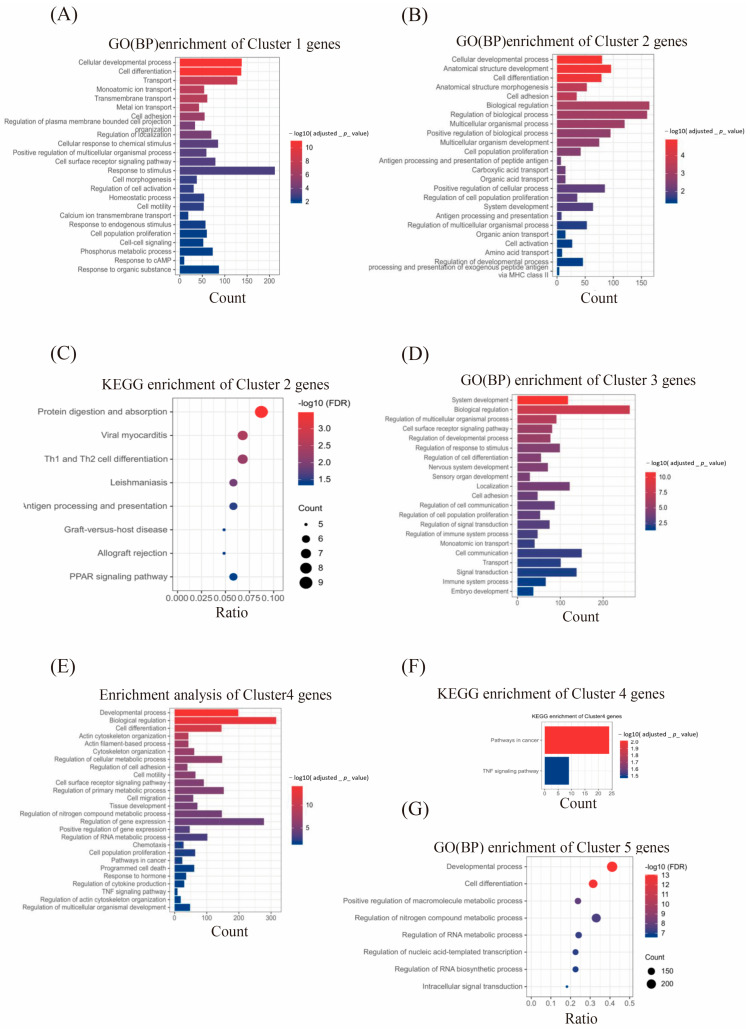
Biological processes involved in the DEGs. (**A**) GO (Biological Process, BP) enrichment of Cluster 1 genes. (**B**) GO (Biological Process, BP) enrichment of Cluster 2 genes. (**C**) KEGG enrichment of Cluster 2 genes. (**D**) GO (Biological Process, BP) enrichment of Cluster 3 genes. (**E**) Enrichment analysis of Cluster 4 genes. (**F**) KEGG enrichment of Cluster 4 genes. (**G**) GO enrichment of Cluster 5 genes.

To identify biological processes involved in differentially expressed genes (DEGs), GO and KEGG analyses were performed. Cluster 1 genes (highly expressed in 10^−5^ M MT group) were enriched in cell differentiation, ion transport, cell adhesion, and cell proliferation (Figure 7A), indicating enhanced cellular activity with melatonin supplementation. Cluster 2 genes (lowly expressed in 0 M MT group) were related to developmental processes, metabolism, and immune responses, including Th1 and Th2 cell differentiation and PPAR signaling pathway (Figure 7B,C), suggesting that melatonin alleviates metabolic and immune disorders caused by vitrification. Cluster 3 genes were associated with cell communication and embryonic development (Figure 7D). Clusters 4 and 5 genes (both highly expressed in 0 M MT group) were enriched in cytoskeleton organization, gene expression regulation, and programmed cell death, with KEGG pathways including “Pathways in cancer” and “TNF signaling pathway” (Figure 7E–G). These results indicate that cryopreservation impairs metabolic processes, cytoskeleton integrity, and gene expression, while melatonin supplementation protects against these damages and improves cellular activities.

### 3.8. Differential Transcription Factors Regulating Gene Expression in Vitrified and Thawed Mouse Embryos

**Figure 8:**
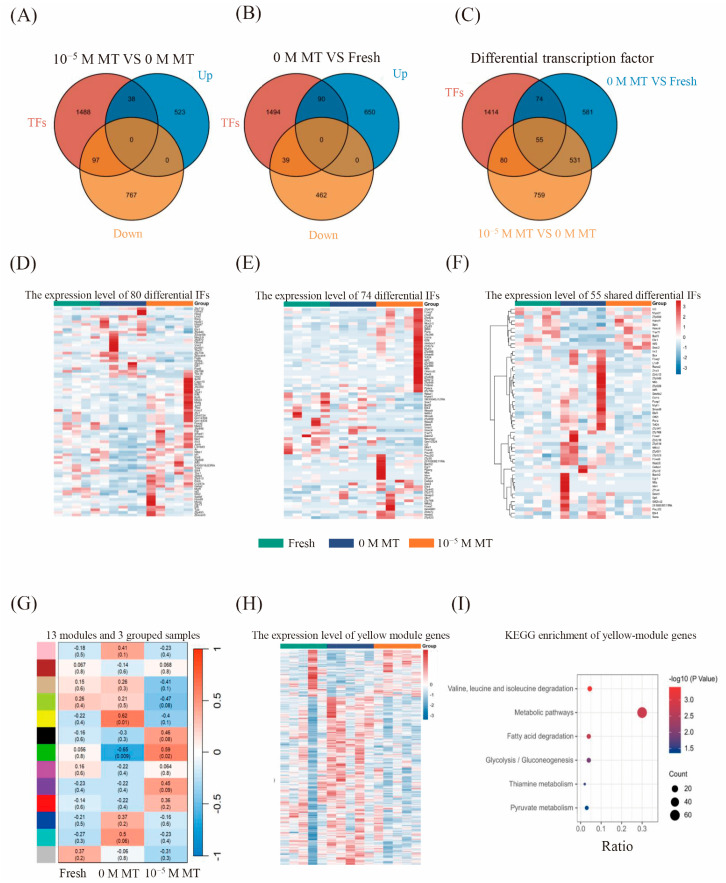
Differential transcription factors regulating gene expression in vitrified and thawed mouse embryos. (**A**–**C**) Venn diagrams. (**D**–**F**) Heatmaps showing the expression levels of differential TFs in different groups. (**G**) Heatmap of the correlation of the 13 modules with the three subgroup samples. Each row corresponds to a co-expression module, and the number in each square represents the correlation coefficient of the module with the developmental stage, with *p*-values in parentheses. Red and blue squares indicate positive and negative correlations, respectively; white indicates no correlation. (**H**) Heatmap showing the expression levels of 420 genes in the ME yellow module. Colours represent log2-transformed multiplicity of differences; redder colours represent greater up-regulated multiplicity and bluer colours represent greater down-regulated multiplicity. (**I**) Bubble chart showing the KEGG pathways enriched by the genes expressed in the ME yellow module. The horizontal coordinate is the ratio of the number of target genes enriched to the target pathways to the total number of target genes, vertical coordinate represents the six KEGG pathways enriched.

To identify transcription factors (TFs) regulating vitrified embryo development, 237 differential TFs were screened using the AnimalTFDB database. Compared with the 0 M MT group, the 10^−5^ M MT group contained 38 upregulated and 97 downregulated TFs (Figure 8A), 90 upregulated and 39 downregulated TFs compared with the Fresh group (Figure 8B), and shared 55 common differential TFs identified (Figure 8C). Heatmaps visualized TF expression patterns across groups (Figure 8D–F). Notably, the expression of pluripotency genes, including *Dmrt1*, *Sox4*, *Sox7*, and *Pou2f2*, was elevated in the 10^−5^ M MT group versus the 0 M MT group, but still lower than that in embryos of the Fresh group. Heatmaps show the expression levels of 420 genes in the ME yellow module (Figure 8G,H), whereas a bubble chart shows the KEGG pathways enriched by the genes expressed in the ME yellow module (Figure 8I). These results indicate that vitrification affects pluripotency gene expression, thereby influencing embryonic developmental quality, and melatonin could reverse these defects.

### 3.9. Effects of Melatonin on the Molecular Network in Alleviating Cryoinjury in Mouse Embryos

**Figure 9:**
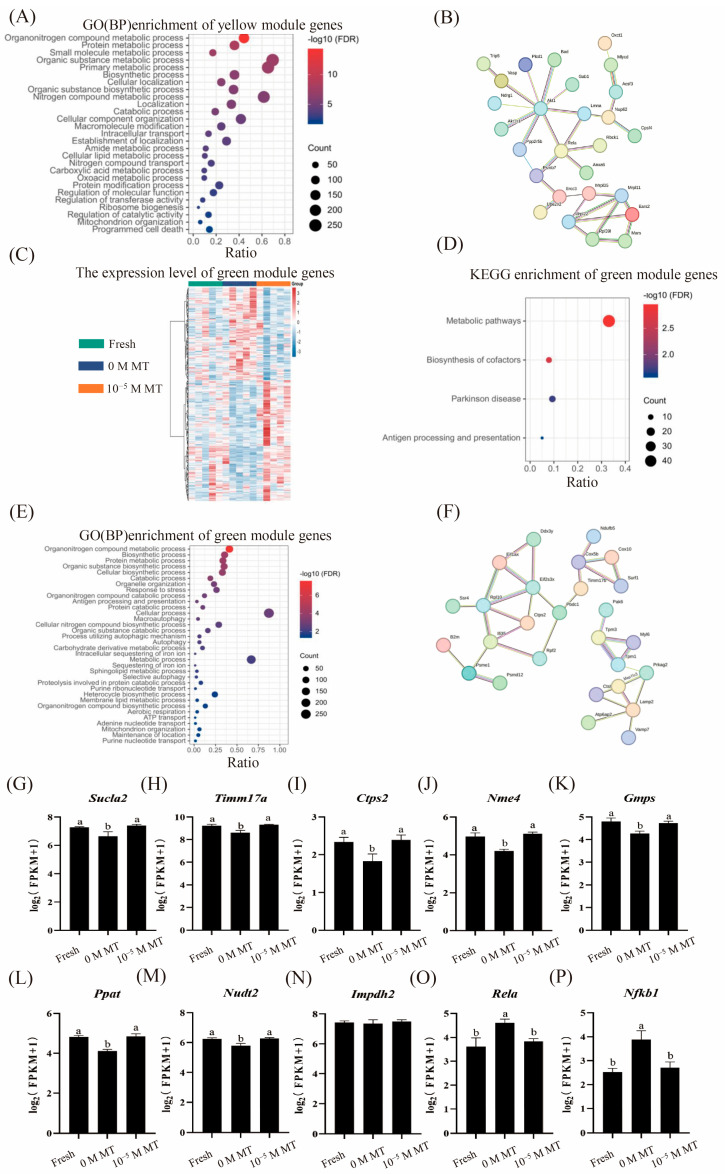
Effects of melatonin on the molecular network and expression of key embryonic genes in vitrified mouse embryos. (**A**) Bubble chart showing the GO terms enriched by the genes expressed in the ME yellow module. (**B**) Protein–protein interaction (PPI) network diagram of hub genes. (**C**) Heatmap showing the expression levels of 334 genes. (**D**) Bubble chart showing the KEGG pathways enriched. (**E**) Bubble chart showing the GO terms. (**F**) PPI network diagram of hub genes. (**G**–**P**) The relative mRNA expression levels, represented as log_2_(FPKM + 1): (**G**) Sucla2, (**H**) Timm17α, (**I**) Ctps2, (**J**) Nme4, (**K**) Gmps, (**L**) Ppat, (**M**) Nudt2, (**N**) Impdh2, (**O**) Rela, (**P**) Nfkb1. Different letters represent statistically significant differences (*p* < 0.05).

To further investigate the function of the DEGs, the pathways related to programmed cell death, mitochondrial organization, catalytic and transferase activity regulation, and metabolic processes, including fatty acids, pyruvate, amino acids, and proteins, were enriched (Figure 9A). These results indicate that vitrification affects macromolecular metabolism, mitochondrial activity, and enzyme function in mouse embryos. Network analysis of 86 hub genes revealed 85 protein nodes and 40 edges, Among hub genes in co-expression network, *Rela* involved in apoptosis and mitochondrial regulation, confirming programmed cell death in the vitrified embryos (Figure 9B). Meanwhile, 334 genes (Figure 9C) were enriched across “Biosynthesis of cofactors,” “Metabolic pathways,” “Parkinson disease,” and “Antigen processing and presentation” (Figure 9D), alongside biological processes including protein metabolism, autophagy, sphingolipid metabolism, aerobic respiration, ATP transport, and mitochondrial organization (Figure 9E). These results indicate that melatonin affects cellular metabolism, mitochondrial activity, and macromolecular synthesis to alleviate cryoinjury. Screening hub genes (|KME| ≥ 0.8) and constructing PPI networks yielded 97 hub genes with 94 protein nodes and 40 edges (Figure 9F). The core gene *Ctps2*, a rate-limiting enzyme in cytosine nucleotide synthesis, was downregulated after vitrification but rescued by melatonin, indicating that melatonin promotes recovery of embryonic metabolic processes. Furthermore, mitochondrial genes *Sucla2* and *Timm17a* showed significantly increased expression in the 10^−5^ M MT group versus the 0 M MT group (*p* < 0.05) (Figure 9G,H), consistent with improved mitochondrial membrane potential and ATP levels, confirming melatonin enhances mitochondrial function and embryonic development. Vitrification downregulated nucleotide synthesis genes, including *Ctps2*, *Nme4*, *Gmps*, *Ppat*, *Nudt2*, and *Impdh2* (Figure 9I–N), while melatonin increased their expression, promoting nucleotide synthesis. NF-κB pathway genes *Rela* and *Nfkb1* were upregulated after cryopreservation and alleviated by melatonin (Figure 9O,P), suggesting melatonin regulates post-thaw gene transcription through the NF-κB pathway.

## 4. Discussion

Embryo vitrification serves as an indispensable method for modern assisted reproduction techniques and genetic resource preservation, enabling indefinite preservation of germplasm, facilitating global distribution of elite genetics, and significantly enhancing livestock breeding programs. In recent years, embryo vitrification has advanced considerably; however, the post-thaw developmental competence remains inferior to that of fresh embryos. Vitrification achieves favorable survival, implantation, and delivery rates in human assisted reproduction, but requires high cryoprotectant concentrations and rapid cooling to prevent ice crystal formation [15,16]. The vitrification process induces lipid peroxidation, oxidative stress sensitivity, membrane damage, and structural disruption, compromising post-thaw implantation and developmental potential [21,22]. Current optimization strategies focus on increasing heating rates, enhancing solution viscosity, and reducing sample volume to prevent recrystallization and organelle damage during thawing. Nevertheless, the impacts of vitrification on subsequent embryonic development remain incompletely resolved.

In the present study, supplementing the vitrification and thawing media with melatonin at 10^−5^ M improved embryo development and the live birth rate following embryo transfer. The current results demonstrate that melatonin promotes post-thaw embryonic development through its antioxidant function, which reduces ROS production, enhances mitochondrial membrane potential and ATP synthesis, and consequently improves ICM pluripotency and developmental competence. These findings align with previous reports demonstrating that melatonin improves vitrified oocyte developmental competence by efficiently scavenging oxygen free radicals, maintaining membrane permeability, and protecting mitochondrial function [17,23]. Melatonin also reduces the ROS accumulation that damages DNA, lipids, proteins, and cellular membranes, and upregulates aquaporin genes to regulate intracellular water and solute exchange in vitrified oocytes [24]. These functions of melatonin could protect the embryo from damage induced during the vitrification process.

During early embryonic development, mitochondria serve as critical organelles supplying ATP through oxidative phosphorylation for most cellular processes. Mitochondrial numbers increase substantially during embryogenesis to meet energy demands for blastocyst formation; consequently, mitochondrial dysfunction can precipitate developmental arrest [25,26]. Mitochondrial membrane potential is intimately associated with apoptotic regulation, as its reduction represents a pivotal event in cell death pathways. Melatonin exerts significant effects on mitochondrial function by improving electron transport chain efficiency, increasing ATP production, and reducing oxidative damage [27,28]. Our observation that melatonin elevates ATP levels in frozen embryos corroborates previous findings. Furthermore, embryos treated with melatonin exhibited higher MMP, indicating enhanced anti-apoptotic capacity. Notably, some studies report that melatonin can reduce mitochondrial membrane potential, induce respiratory quiescence and maintain low metabolic states as potential compensatory mechanisms for energy deficiency [29,30,31,32]; thus, melatonin could protect embryos from unfavorable conditions. Oxidative stress impairs embryo quality by causing oxidative damage, which can be reduced by melatonin. Our findings demonstrate that cryoinjury elevates embryonic ROS levels, while melatonin supplementation significantly reduces oxidative damage. Previous studies also confirmed similar outcomes from melatonin in elevating ICM cell number in embryos [32,33]. Melatonin supplementation promotes post-thaw embryonic development, increases ICM-to-total cell ratios, and positively influences transferred embryo development. The underlying mechanism likely involves melatonin-mediated enhancement of ICM pluripotency maintenance and developmental competence through improved mitochondrial function and reduced oxidative damage.

The molecular networks by which vitrification impairs embryonic development and how melatonin exerts protective effects at the transcriptional level were elucidated in the current study. Gene expression analysis revealed that vitrification-associated genes were predominantly enriched in pathways, including valine, leucine, and isoleucine degradation; metabolic pathways; fatty acid degradation; glycolysis/gluconeogenesis; thiamine metabolism; and pyruvate metabolism. These results indicate that vitrification disrupts embryonic metabolic processes and compromises subsequent developmental potential. Further screening of the gene regulatory network identified *Rela* as a central hub in the co-expression network. *Rela* participates in NF-κB signal transduction, and impairment of this pathway can trigger extensive programmed cell death across multiple tissues during mouse embryonic development [34,35]. Following vitrification, *Rela* and *Nfkb1* expression levels were significantly upregulated in mouse blastocysts, indicating excessive NF-κB pathway activation. Consistent with this finding, a previous study demonstrated NF-κB signaling upregulation in vitrified mouse blastocysts, leading to pro-inflammatory cytokine release (e.g., IL-6) seven days post-transfer, an effect ameliorated by resveratrol treatment [36]. As a potent antioxidant, melatonin also inhibited excessive *Rela* and *Nfkb1* activation. Previous investigations have established melatonin’s involvement in numerous metabolic pathways during embryonic development, including regulation of lipid metabolism through cumulus cell receptors to promote oocyte maturation [37,38]. Collectively, these findings demonstrate that vitrification disrupts metabolic homeostasis and activates stress-responsive pathways such as NF-κB signaling, while melatonin supplementation effectively attenuates these molecular perturbations, thereby preserving embryonic developmental competence.

## 5. Conclusions

Supplementation of melatonin in vitrification and thawing media promotes post-thaw embryonic development and outcomes following embryo transfer. The underlying mechanism involves the melatonin-mediated enhancement of ICM pluripotency maintenance and developmental competence through improved mitochondrial function and reduced oxidative damage. Additionally, melatonin improved the expression of mitochondrial function-related genes *Sucla2* and *Timm17a* in embryos post-vitrification and thawing. Therefore, the current results provide a high efficiency vitrification and thawing method by melatonin supplementation.

## Figures and Tables

**Table 1:** Reaction solution.

Component	Dose (μL)
Click Reaction Buffer	430
Click Additive	50
Azide 594	1
CuSO_4_	20

## Data Availability

The raw smart-seq 2 data were deposited in the Genome Sequence Archive (GSA) (Accession number: CRA041682), which is publicly accessible at https://ngdc.cncb.ac.cn/gsa (accessed on 1 April 2026). Further inquiries can be directed to the corresponding authors.

## Supplementary Materials

The following supporting information can be downloaded at: https://www.mdpi.com/article/10.3390/antiox15060667/s1, Figure S1: Standard curve.
