# Supplementary figures and images for: Melatonin Mitigates Vitrification-Induced Cryoinjury in Mouse Embryos by Alleviating Metabolic Alterations
Source: Antioxidants (Basel). 2026 May 26;15(6):667. doi: 10.3390/antiox15060667 (PMC13295670; doi:10.3390/antiox15060667)

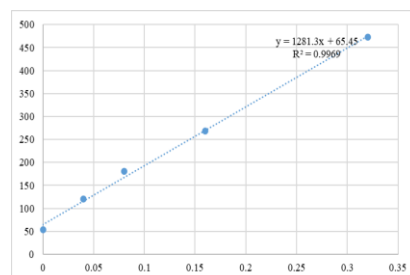

**Figure S1.** Standard curve.

Supplement: Supplementary file 1 [file antioxidants-15-00667-s001.zip › antioxidants-4277600-supplementary.pdf]
